# Supplementary material for: Objective Quantification of In-Hospital Patient Mobilization after Cardiac Surgery Using Accelerometers: Selection, Use, and Analysis
Source: Sensors (Basel). 2021 Mar 11;21(6):1979. doi: 10.3390/s21061979 (PMC7999757; doi:10.3390/s21061979)
Supplement: Supplementary file 1 [file sensors-21-01979-s001.zip › Supplementary Figure S1.pdf]

Supplementary Figure S1 for the Sensors manuscript:

Objective quantification of in-hospital patient mobilization after cardiac surgery using accelerometers: Selection, Use, and Analysis

Correspondence: f.halfwerk@mst.nl

CONSORT
TRANSPARENT REPORTING of TRIALS

CONSORT 2010 Flow Diagram – MOVEMENTT study

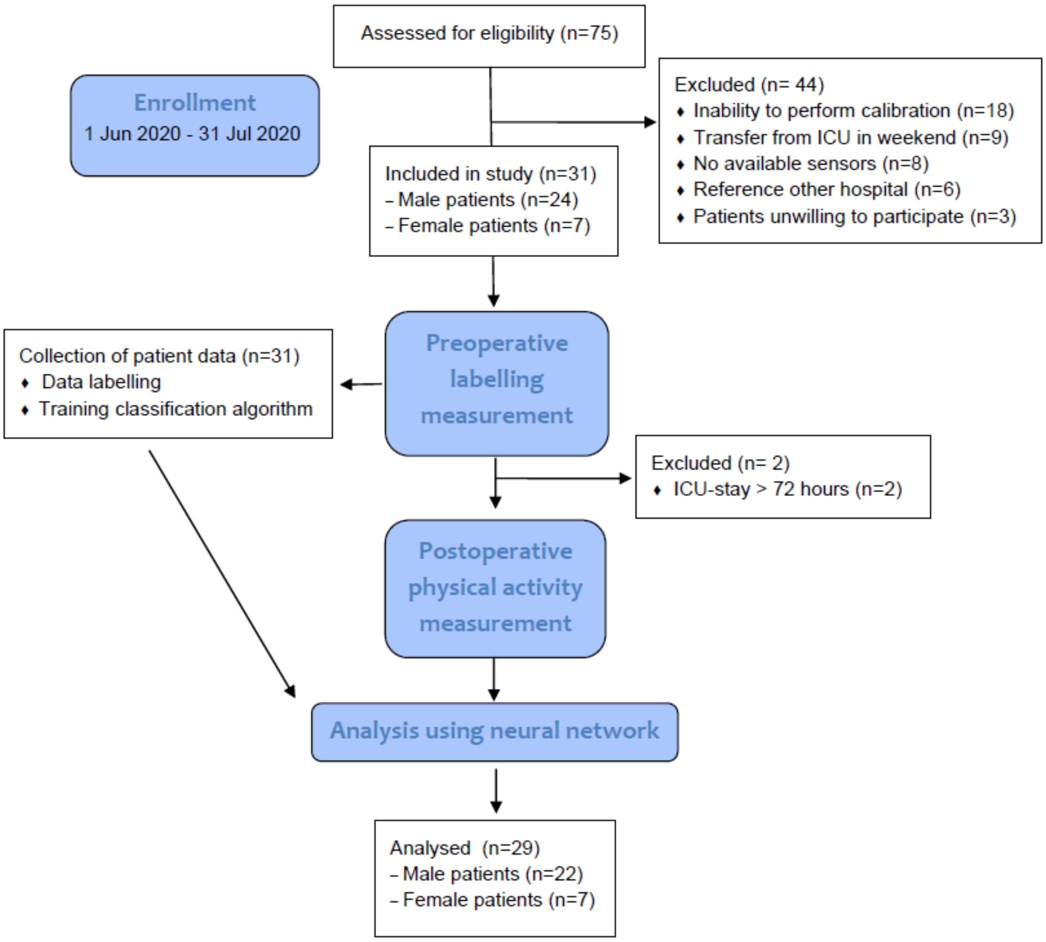

Supplementary Figure S1. Study flow diagram according to CONSORT [1]

Reference

1. Schulz, K.F.; Altman, D.G.; Moher, D.; Group, C. CONSORT 2010 statement: updated guidelines for reporting parallel group randomised trials. BMJ **2010**, 340, c332, doi:10.1136/bmj.c332.
