# Supplementary material for: Objective Quantification of In-Hospital Patient Mobilization after Cardiac Surgery Using Accelerometers: Selection, Use, and Analysis
Source: Sensors (Basel). 2021 Mar 11;21(6):1979. doi: 10.3390/s21061979 (PMC7999757; doi:10.3390/s21061979)
Supplement: Supplementary file 1 [file sensors-21-01979-s001.zip › Supplementary Figure S2 and S3.docx]

Supplementary Figures S2 and S3

Objective quantification of in-hospital patient mobilization after cardiac surgery using accelerometers: Selection, Use, and Analysis

Frank R. Halfwerk ^1, 2, *^, Jeroen H.L. van Haaren ^1^, Randy Klaassen ^3^, Robby W. van Delden ^3^, Peter H. Veltink ^4^ and Jan G. Grandjean ^1, 2^

| **Citation:** Halfwerk, F.R. et al. *Sensors* **2021**, *21*, x. https://doi.org/10.3390/xxxxx  Received: date  Accepted: date  Published: date  **Publisher’s Note:** MDPI stays neutral with regard to jurisdictional claims in published maps and institutional affiliations.  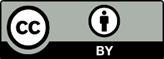  **Copyright:** © 2021 by the authors. Submitted for possible open access publication under the terms and conditions of the Creative Commons Attribution (CC BY) license (http://creativecommons.org/licenses/by/4.0/). |
| --- |

^1^ Thoraxcentrum Twente, Medisch Spectrum Twente, PO Box 50 000, 7500 KA Enschede,

The Netherlands; [f.halfwerk@mst.nl](mailto:f.halfwerk@mst.nl), [j.grandjean@mst.nl](mailto:j.grandjean@mst.nl), [j.vanhaaren@mst.nl](mailto:j.vanhaaren@mst.nl)

^2^ Dept. of Biomechanical Engineering, TechMed Centre, University of Twente, PO Box 217, 7500 AE Enschede, The Netherlands

^3^ Human Media Interaction Lab, University of Twente, PO Box 217, 7500 AE Enschede, The Netherlands; [r.klaassen@utwente.nl](mailto:r.klaassen@utwente.nl), [r.w.vandelden@utwente.nl](mailto:r.w.vandelden@utwente.nl)

^4^ Dept. of Biomedical Signals and Systems, Faculty of Electrical Engineering, Mathematics and Computer Science, University of Twente, PO Box 217, 7500 AE Enschede, The Netherlands; [p.h.veltink@utwente.nl](mailto:p.h.veltink@utwente.nl)

* Correspondence: [f.halfwerk@mst.nl](mailto:f.halfwerk@mst.nl)

| **** |
| --- |
| **Supplementary Figure S2.** Overall neural network accuracy using n number of patients (starting from n = 2) in training data set. Accuracy was determined by averaging results from Leave-One-Out (LOO) validation over n number of patients. All patients (black line), and male (blue line) and female (red line) showed an accuracy around 95% starting from n = 2.  Vertical lines depict number of patients included for the entire study group (n = 31), and subgroups male (n = 24) and female (n = 7) patients. The network accuracy was calculated before excluding two patients with prolonged intensive care unit (ICU) stay. |

Supplementary Figure S2

Supplementary Figure S3

|  |  |
| --- | --- |
| (**a**) | (**b**) |
|  |  |
| (**c**) | (**d**) |
|  |  |
| (**e**) | (**f**) |

**Supplementary Figure S3.** Neural network recall and precision per activity using n number of patients (starting from n = 2) in training data set. Precision and recall were determined by averaging results from LOO cross-validation over n number of patients. All patients (black line), and male (blue line) and female (red line) showed an accuracy exceeding 78% starting from n = 2. (**a**) Lying in bed; (**b**) Sitting in a chair; (**c**) Standing; (**d**) Walking; (**e**) Cycling; (**f**) Walking the stairs.

Vertical lines depict number of patients included for the entire study group (n = 31), and subgroups male (n = 24) and female (n = 7) patients. The network accuracy was calculated before excluding two patients with prolonged ICU stay.
