# Supplementary material for: Objective Quantification of In-Hospital Patient Mobilization after Cardiac Surgery Using Accelerometers: Selection, Use, and Analysis
Source: Sensors (Basel). 2021 Mar 11;21(6):1979. doi: 10.3390/s21061979 (PMC7999757; doi:10.3390/s21061979)
Supplement: Supplementary file 1 [file sensors-21-01979-s001.zip › Supplementary File 1.docx]

Supplementary File 1 – Neural network training protocol

Objective quantification of in-hospital patient mobilization after cardiac surgery using accelerometers: Selection, Use, and Analysis

Frank R. Halfwerk ^1, 2, *^, Jeroen H.L. van Haaren ^1^, Randy Klaassen ^3^, Robby W. van Delden ^3^, Peter H. Veltink ^4^ and Jan G. Grandjean ^1, 2^

| **Citation:** Halfwerk, F.R. et al. *Sensors* **2021**, *21*, x. https://doi.org/10.3390/xxxxx  Received: date  Accepted: date  Published: date  **Publisher’s Note:** MDPI stays neutral with regard to jurisdictional claims in published maps and institutional affiliations.  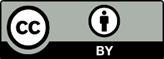  **Copyright:** © 2021 by the authors. Submitted for possible open access publication under the terms and conditions of the Creative Commons Attribution (CC BY) license (http://creativecommons.org/licenses/by/4.0/). |
| --- |

^1^ Thoraxcentrum Twente, Medisch Spectrum Twente, PO Box 50 000, 7500 KA Enschede,

The Netherlands; [f.halfwerk@mst.nl](mailto:f.halfwerk@mst.nl), [j.grandjean@mst.nl](mailto:j.grandjean@mst.nl), [j.vanhaaren@mst.nl](mailto:j.vanhaaren@mst.nl)

^2^ Dept. of Biomechanical Engineering, TechMed Centre, University of Twente, PO Box 217, 7500 AE Enschede, The Netherlands

^3^ Human Media Interaction Lab, University of Twente, PO Box 217, 7500 AE Enschede, The Netherlands; [r.klaassen@utwente.nl](mailto:r.klaassen@utwente.nl), [r.w.vandelden@utwente.nl](mailto:r.w.vandelden@utwente.nl)

^4^ Dept. of Biomedical Signals and Systems, Faculty of Electrical Engineering, Mathematics and Computer Science, University of Twente, PO Box 217, 7500 AE Enschede, The Netherlands; [p.h.veltink@utwente.nl](mailto:p.h.veltink@utwente.nl)

* Correspondence: [f.halfwerk@mst.nl](mailto:f.halfwerk@mst.nl)

Supplementary File 1 – Neural network training protocol

The neural network was trained using labelled data from 31 patients (24 male and 7 female patients) on hospital admission, one day before cardiac surgery. After signing informed consent, patients performed static and dynamic activities according a fixed protocol.

Patients were asked to subsequently stand, sit on a chair, lie on a bench on the back, right and left side. Each posture was held for 30 seconds. Transition time between posture was approximately 10 seconds. Afterwards, patients walked back and forth on the corridor (10 meters), cycled on an exercise bike and took one flight of stairs up and down (15 steps). The dynamic activities were performed for a minimal duration of 30 seconds and were alternated with 10 seconds of standing still. The training protocol also contained short parts of free movement, e.g. walking from the patient room to the corridor. For each patient, total duration of the exercises was 10 to 15 minutes.

For each patient, an average of eleven minutes of labelled data was used. Segments of measured data were manually labelled by a researcher as lying, sitting, standing, walking, cycling or walking the stairs with hard coding. The measured data was plotted with Matlab (see Figure 1 in this Supplementary File 1). With visual inspection of the plots, data samples were labelled as one of the six activities.

| 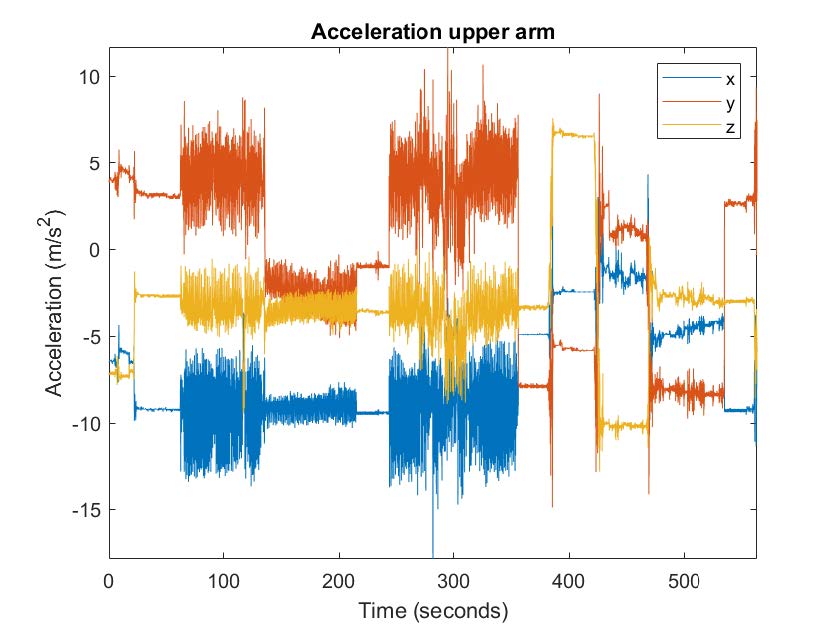 | 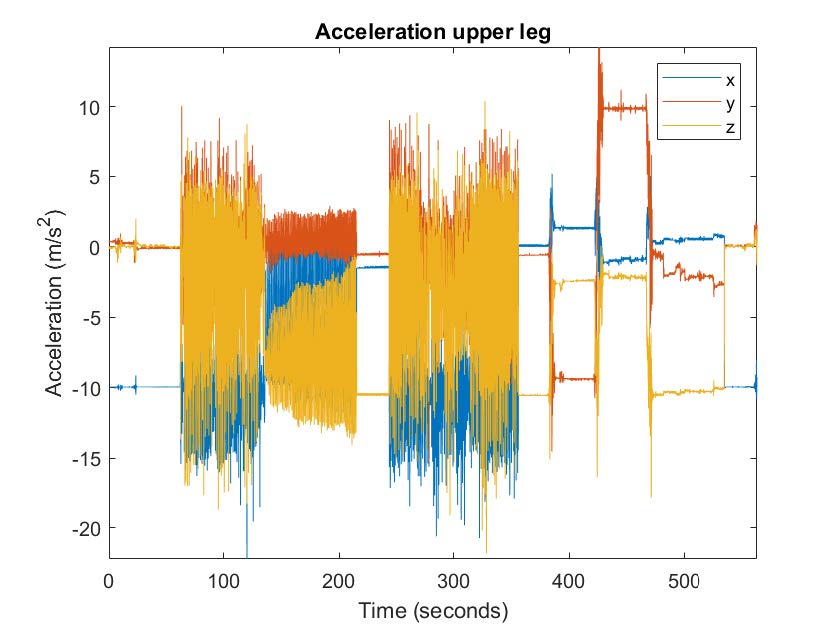 |
| --- | --- |
| (**a**) | (**b**) |
| 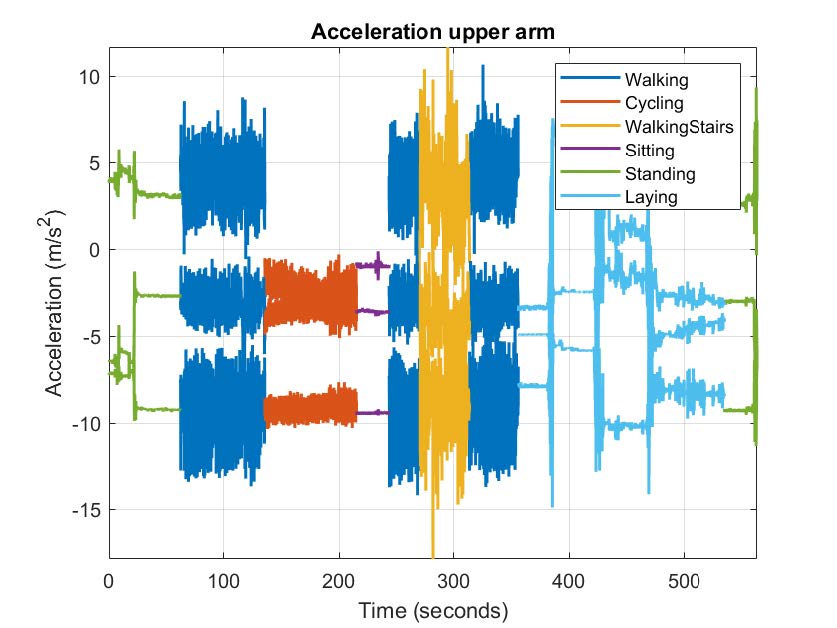 | 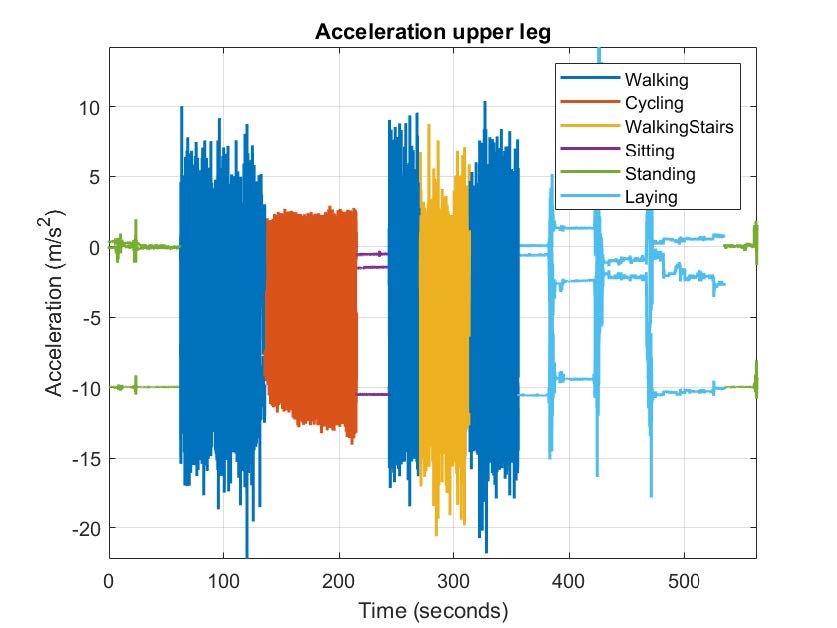 |
| (**c**) | (**d**) |

**Supplementary File 1, Figure 1.** Accelerometer data captured with the AX3 sensor attached to the upper arm (**a**) or upper leg (**b**). Data was labelled by the researcher for both positions (**c, d**).
