# Supplementary material for: Objective Quantification of In-Hospital Patient Mobilization after Cardiac Surgery Using Accelerometers: Selection, Use, and Analysis
Source: Sensors (Basel). 2021 Mar 11;21(6):1979. doi: 10.3390/s21061979 (PMC7999757; doi:10.3390/s21061979)
Supplement: Supplementary file 1 [file sensors-21-01979-s001.zip › Supplementary Tables S1 and S2.docx]

Supplementary Tables S1 and S2

Objective quantification of in-hospital patient mobilization after cardiac surgery using accelerometers: Selection, Use, and Analysis

Frank R. Halfwerk ^1, 2, *^, Jeroen H.L. van Haaren ^1^, Randy Klaassen ^3^, Robby W. van Delden ^3^, Peter H. Veltink ^4^ and Jan G. Grandjean ^1, 2^

| **Citation:** Halfwerk, F.R. et al. *Sensors* **2021**, *21*, x. https://doi.org/10.3390/xxxxx  Received: date  Accepted: date  Published: date  **Publisher’s Note:** MDPI stays neutral with regard to jurisdictional claims in published maps and institutional affiliations.  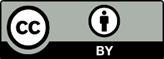  **Copyright:** © 2021 by the authors. Submitted for possible open access publication under the terms and conditions of the Creative Commons Attribution (CC BY) license (http://creativecommons.org/licenses/by/4.0/). |
| --- |

^1^ Thoraxcentrum Twente, Medisch Spectrum Twente, PO Box 50 000, 7500 KA Enschede,

The Netherlands; [f.halfwerk@mst.nl](mailto:f.halfwerk@mst.nl), [j.grandjean@mst.nl](mailto:j.grandjean@mst.nl), [j.vanhaaren@mst.nl](mailto:j.vanhaaren@mst.nl)

^2^ Dept. of Biomechanical Engineering, TechMed Centre, University of Twente, PO Box 217, 7500 AE Enschede, The Netherlands

^3^ Human Media Interaction Lab, University of Twente, PO Box 217, 7500 AE Enschede, The Netherlands; [r.klaassen@utwente.nl](mailto:r.klaassen@utwente.nl), [r.w.vandelden@utwente.nl](mailto:r.w.vandelden@utwente.nl)

^4^ Dept. of Biomedical Signals and Systems, Faculty of Electrical Engineering, Mathematics and Computer Science, University of Twente, PO Box 217, 7500 AE Enschede, The Netherlands; [p.h.veltink@utwente.nl](mailto:p.h.veltink@utwente.nl)

* Correspondence: [f.halfwerk@mst.nl](mailto:f.halfwerk@mst.nl)

This supplement contains two supplementary Tables:

- Supplementary Table S1 with technical and clinical requirements for devices used to classify inpatient physical activity after cardiac surgery
- Supplementary Table S2 with application of technical and clinical requirements on 18 wearable devices

Supplementary Table S1

**Table S1.1** Technical requirements for a device to classify inpatient physical activities after cardiac surgery as determined from literature study and stakeholders interviews, ranked from highest to lowest importance.

|  | **Technical requirements** | **Considerations** |
| --- | --- | --- |
|  | Classification of various activities   - Static: Lying, sitting, standing; - Dynamic: Walking, cycling, walking the stairs. | In an average hospital stay of 5 to 7 days, patient activities change from lying in bed and sitting in a chair, towards walking on the ward, cycling on an exercise bike and walking the stairs before discharge from the hospital. |
|  | Sampling rate between 60 – 100 Hz | Body movements have a bandwidth of 10 Hz. For movement analysis, raw signals need to be sampled with 60 to 100 Hz. |
|  | Battery life for measuring up to 7 consecutive days | Patients have an average hospital stay of 5 to 7 days after cardiac surgery. It would be inconvenient if measurements are interrupted by charging or removing the device for patients (patient comfort, privacy) and professionals (loss of information). |
|  | Local data storage up to 7 consecutive days | Patients have an average hospital stay of 5 to 7 days after cardiac surgery. As measurements should not be interrupted, physical data export is unwanted. |
|  | Data extraction from the device | For (statistical) data analysis and classification, the raw data must be available to develop proprietary algorithms for activity classification. |

**Table S1.2** Clinical requirements for a device to classify inpatient physical activities after cardiac surgery as determined from literature study and stakeholders interviews, ranked from highest to lowest importance.

|  | **Clinical requirements** | **Considerations** |
| --- | --- | --- |
|  | **Patient related** |  |
|  | No burden during hospital stay and no hindrance in mobilization | Patient discomfort is undesirable. Hindrance would negatively effect actual mobilization and reduce accuracy. |
|  | Minimal soft tissue deformation | For patient comfort, the device should not cause damage or pain to the patient. |
|  | Device placement away from wound area | Wound healing and wound care must not be compromised with respect to potential wound infections. Cardiac surgery is often performed with a median sternotomy of thoracotomy with chest drains placed under the rib cage. CABG patients have a wound on their forearm or internal side of a leg for graft harvesting. |
|  | Meeting regulations regarding patient privacy and integrity | A clinical trial can only be conducted with patient permission, ethical approval and is thus subjected to GDPR, hospital and university regulations. |
|  | **Practical and implementation** |  |
|  | No impediment in daily nursing care | Quality of regular patient care should not be compromised. Adaptations to perform daily care, such as taking of the device for showering could cause data inconsistencies or data loss and is unwanted. |
|  | Device is CE certified | The clinical pilot can only be performed in the hospital when a device is CE certified. |
|  | Total costs for clinical pilot study should not exceed €3,000 | A budget of €3,000 from Stichting Hartcentrum Twente was obtained for this study and should cover total project costs. |
|  | Data analysis is attainable with supplied software and/or is available within University license | The need of separate software could be costly and inconvenient to use in a hospital digital environment. This could potentially slow the process of data analysis |

CABG = Coronary artery bypass grafting; CE = Conformité Européenne marking; GDPR = European Union General Data Protection Regulation; ICU = Intensive care unit

Supplementary Table S2

**Table S2** Requirements for a device to classify inpatient physical activities after cardiac surgery applied on 18 wearable devices. The devices are ordered from high to low on meeting the requirements.

|  | **Requirements** | **AX3** | **ActiGraph** | **MOX1** | **MoveMonitor** | **Fibion** | **Activ8** |
| --- | --- | --- | --- | --- | --- | --- | --- |
|  | **Technical** |  |  |  |  |  |  |
|  | Lying  Sitting  Standing  Walking  Cycling  Walking the stairs | √*  √*  √  √  √  √ | √*  √*  √  √  √  √ | √*  √*  √  √  √  √ | √  X  √  √  X  X | X  X  √  √  √  X | X  X  √  √  √  X |
|  | Sampling rate 60 – 100 Hz | √ | √ | √ | √ | X | X |
|  | Battery life ≥ 5 days | √ | √ | X | √ | √ | √ |
|  | Local data storage ≥ 5 days | √ | √ | √ | √ | √ | √ |
|  | Data extraction | √ | √ | √ | √ | √ | √ |
|  | **Clinical** |  |  |  |  |  |  |
|  | No discomfort and hindrance in mobilization | √ | √ | √ | √ | √ | √ |
|  | No tissue deformation | √ | √ | √ | X^†^ | √ | √ |
|  | Device placement outside wound area | √ | √ | √ | √ | √ | √ |
|  | Patient privacy secured | √ | √ | √ | √ | √ | √ |
|  | No impediment of daily patient care | √ | X | √ | √ | X | X |
|  | CE certification | √ | √ | √ | √ | √ | ? |
|  | Costs pilot study ≤ €3,000 | √ | X | √ | X | √ | √ |
|  | Analysis software available | √ | √ | √ | √ | √ | √ |

√ = device meets requirement; X = device does not meet requirement; ? = unknown; * = Classification of lying and sitting only expected with two sensor measurements; ^†^ = Stress on lower back when lying supine due to sensor location

**Table S2** (Continued)

|  | **Requirements** | **activPAL** | **VitalPatch** | **Health Watch** | **SenseWear Pro3** |
| --- | --- | --- | --- | --- | --- |
|  | **Technical** |  |  |  |  |
|  | Lying  Sitting  Standing  Walking  Cycling  Walking the stairs | X  X  √  √  X  X | X  X  √  X  X  X | X  X  X  √  X  X | X  X  X  √  X  X |
|  | Sampling rate 60 – 100 Hz | X | √ | ? | √ |
|  | Battery life ≥ 5 days | √ | √ | X | √ |
|  | Local data storage ≥ 5 days | √ | X | √ | ? |
|  | Data extraction | √ | √ | √ | √ |
|  | **Clinical** |  |  |  |  |
|  | No discomfort and hindrance in mobilization | √ | √ | √ | √ |
|  | No tissue deformation | √ | √ | √ | ? |
|  | Device placement outside wound area | √ | X | √ | √ |
|  | Patient privacy secured | √ | √ | √ | √ |
|  | No impediment of daily patient care | X | X | X | X |
|  | CE certification | √ | √ | √ | ? |
|  | Costs pilot study ≤ €3,000 | X | ? | √ | X |
|  | Analysis software available | √ | ? | √ | √ |

√ = device meets requirement; X = device does not meet requirement; ? = unknown;

**Table S2** (Continued)

|  | **Requirements** | **HeartGuide** | **Fitbit** | **Garmin VivoSmart** | **Alvita Ultimate** |
| --- | --- | --- | --- | --- | --- |
|  | **Technical** |  |  |  |  |
|  | Lying  Sitting  Standing  Walking  Cycling  Walking the stairs | X  X  X  √  X  X | X  X  X  √  X  X | X  X  X  √  X  X | X  X  X  √  X  X |
|  | Sampling rate 60 – 100 Hz | ? | ? | ? | ? |
|  | Battery life ≥ 5 days | X | X | √ | √ |
|  | Local data storage ≥ 5 days | √ | √ | √ | √ |
|  | Data extraction | √ | √ | √ | X |
|  | **Clinical** |  |  |  |  |
|  | No discomfort and hindrance in mobilization | √ | √ | √ | √ |
|  | No tissue deformation | √ | √ | √ | √ |
|  | Device placement outside wound area | √ | √ | √ | √ |
|  | Patient privacy secured | √ | √ | √ | √ |
|  | No impediment of daily patient care | X | √ | √ | X |
|  | CE certification | √ | √ | √ | √ |
|  | Costs pilot study ≤ €3,000 | X | √ | √ | √ |
|  | Analysis software available | ? | √ | √ | X |

√ = device meets requirement; X = device does not meet requirement; ? = unknown

**Table S2** (Continued)

|  | **Requirements** | **Motiv Ring** | **Welsense Vu** | **BCGMCU/SCA11H** | **E4 Wristband** |
| --- | --- | --- | --- | --- | --- |
|  | **Technical** |  |  |  |  |
|  | Lying  Sitting  Standing  Walking  Cycling  Walking the stairs | X  X  X  √  X  X | √  X  X  X  X  X | √  X  X  X  X  X | X  X  X  X  X  X |
|  | Sampling rate 60 – 100 Hz | ? | ? | ? | X |
|  | Battery life ≥ 5 days | X | √ | √ | X |
|  | Local data storage ≥ 5 days | √ | ? | √ | X |
|  | Data extraction | ? | ? | √ | √ |
|  | **Clinical** |  |  |  |  |
|  | No discomfort and hindrance in mobilization | √ | √ | √ | √ |
|  | No tissue deformation | √ | √ | √ | √ |
|  | Device placement outside wound area | √ | √ | √ | √ |
|  | Patient privacy secured | √ | √ | √ | √ |
|  | No impediment of daily patient care | √ | √ | √ | X |
|  | CE certification | √ | √ | √ | √ |
|  | Costs pilot study ≤ €3,000 | √ | X | √ | X |
|  | Analysis software available | √ | ? | √ | √ |

√ = device meets requirement; X = device does not meet requirement; ? = unknown.

Data was collected from instructions for use, product websites or peer reviewed academic work. References are available upon request.
